# The prevalence and effects of urinary incontinence in women working in the Universitas Academic Hospital, Bloemfontein 

**DOI:** 10.4102/phcfm.v2i1.99

**Published:** 2010-06-11

**Authors:** Veronique C. Bailey, Meenakshi Bakaya, Siyabulela H. Jada, Kekeletso E. Khalanyane, Wilhelm J. Steinberg, Gina Joubert, Almereau Prollius

**Affiliations:** 1 School of Medicine, University of the Free State, South Africa; 2 Department of Family Medicine, University of the Free State, South Africa; 3 Department of Biostatistics, University of the Free State, South Africa; 4 Department of Obstetrics and Gynaecology, University of the Free State, South Africa

## Abstract

Urinary incontinence affects 30% of women by the time they reach 50 years of age and continues to increase thereafter. Symptoms vary in severity and adversely impact on the physical and psychosocial wellbeing of affected individuals. By means of a self-administered questionnaire, the study investigated the prevalence of urinary incontinence and its effects on the quality of life in women working at the Universitas Academic Hospital in Bloemfontein in 2007. Pregnant women were not included in the study. One hundred and nine questionnaires were analysed. Participants were 24–62 years of age (mean age 44.4 years). Of these, 27.5% reported symptoms of urinary incontinence. Only one affected individual was younger than 30 years. Three-quarters of affected women rated their symptoms as light to moderate. In 34.6% of the affected women, the condition did not interfere with everyday activities at all, but 11.5% reported severe interference. Information regarding urinary incontinence, precautionary measures, such as Kegel exercises, and its associated psychosocial consequences, should be disseminated to women of all ages.

## INTRODUCTION

Urinary incontinence affects women of all ages and is due to the failure of voluntary vesicle and urethral sphincter control, which results in constant or frequent involuntary passage of urine.^1^ In a large community-based study with close to 28 000 participants, Hannestad et al. ^2^ reported a steady increase in the prevalence of urinary incontinence across adulthood until 50 years of age, when prevalence reached 30%. Thereafter, a stabilisation or even slight decline was noted until 75 years of age, at which time prevalence started rising again.^2^ Symptoms vary in nature and severity, being more predominant amongst older people. These may be accompanied by psychological and hygiene-related problems and adversely influence the physical and psychosocial well-being of an affected individual. Different treatment options are available, mostly to minimise the occurrence of incontinence episodes, or limit their impact on everyday life.^1^ 

This study aimed to determine the prevalence of urinary incontinence and its effects on the quality of life of women working at the Universitas Academic Hospital in Bloemfontein in 2007.

Approval to conduct the investigation was granted by the Ethics Committee of the Faculty of Health Sciences, University of the Free State (UFS) and the Clinical Head of the hospital.

## METHOD

The study population consisted of 1714 women working at the Universitas Academic Hospital during June–November 2007. The cleaning staff, employed by a private company, was excluded from the study population.

Two hundred women were selected by means of simple random sample selection from a list of the personnel numbers of female employees obtained from the Human Resources department of the hospital. This number would give fairly precise estimates and would be manageable by the four student researchers. Prospective participants were located individually, informed of the investigation, requested to participate and handed the questionnaire. Illiterate participants consented to waive anonymity, as the questionnaire had to be completed on their behalf.

The ability to speak and/or read Afrikaans, English, Sesotho and/or isiXhosa was required. Pregnant women were excluded from the study.

A pilot study, which included eight female medical students at the UFS, two each for the different questionnaire languages, was performed. The questionnaire was a combination of two questionnaires, namely a Symptom Assessment and a Quality of Life questionnaire. ^3^ Of the initial sample of 200, only 154 qualified for selection at the time of the study. Reasons for non-qualification were:

- being away on leave (15) 
- phantom workers (12) 
- untraceable without contact details (7)
- resignation (6)
- transferred to other hospitals (3)
- study leave (2) 
- deceased (1)

In addition, 35 women refused participation, while 10 more questionnaires were rejected (contradictory feedback or incomplete). Finally, 109 questionnaires were analysed, resulting in a response rate of 70.8% (109/154). Owing to time constraints, replacements were not selected for women who did not qualify for the study.

## RESULTS

The mean age of participants was 44.4 years (range 24–62 years). Thirty (27.5%) participants reported symptoms of urinary incontinence. Only one of 10 women 24–29 years of age had symptoms, compared to 29% in participants > 30 years of age.

Seventy-five per cent of the affected women rated their symptoms as light to moderate, with total scores < 10/21. The median score was 5/21. Incontinence occurred mostly during physical exertion, for example, coughing or sneezing (53.3%) and physical activity/exercise (16.7%). Also, 50% indicated that urinary leakage occurred before they could reach the toilet, while 6.7% ascribed it to having no obvious reason – that leakage occurred ‘all the time’. The standardised questionnaire did not determine the underlying medical conditions, for example, prolapse or detrusor instability.

**FIGURE 1:**
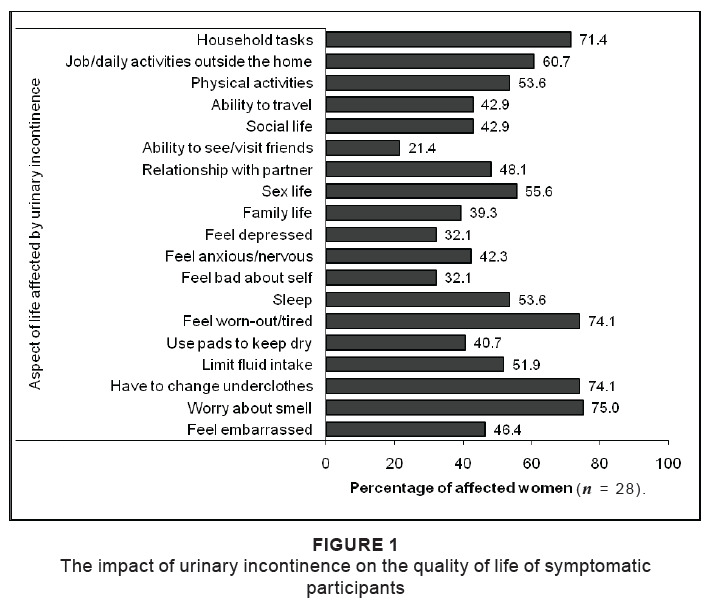
The impact of urinary incontinence on the quality of life of symptomatic participants

On the Quality of Life questionnaire, the affected participants ranked the aspects of life listed in the questionnaire on a scale of 0–10, where 0 = not at all and 10 = a great deal, depending on the extent of their problem (Figure 1). The highest score (median 5/10) was allocated to the use of pads to keep dry. With regard to the extent that symptoms interfered with everyday life from an overall perspective, 34.6% indicated no interference at all, while 11.5% indicated a great extent of interference (median score 2/10). Two women with symptoms of urinary incontinence completed the Quality of Life questionnaire incorrectly.

The prevalence of urinary incontinence amongst these women was 27.5%. Only the incontinence experienced in the preceding 4 weeks was assessed. Should symptoms be investigated over a longer period of time, a higher prevalence might be expected.

## CONCLUSION

The findings confirmed that the quality of life of women affected by urinary incontinence was diminished, with emphasis on concerns about hygiene, social and interpersonal relationships, and self-esteem. Although medical management of incontinence is available, women suffering from urinary incontinence should adopt healthy, non-invasive precautionary measures to prevent the condition, or decrease the severity of symptoms, for example, doing Kegel exercises to strengthen the pelvic floor, increasing dietary fibre intake and reducing alcohol consumption and smoking.

More information regarding urinary incontinence and its psychosocial consequences should be disseminated to women. No literature on similar studies conducted in South Africa could be located. Therefore, a larger multicentre investigation is recommended to promote awareness of incontinence as a medical problem.
